# Potential risk factors and triggers for back pain in children and young adults. A scoping review, part I: incident and episodic back pain

**DOI:** 10.1186/s12998-019-0280-9

**Published:** 2019-11-19

**Authors:** Amber M Beynon, Jeffrey J Hebert, Charlotte Lebouef-Yde, Bruce F Walker

**Affiliations:** 1 College of Science, Health, Engineering and Education, Murdoch University, 90 South Street, Murdoch, Western Australia 6150 Australia; 2 Faculty of Kinesiology, University of New Brunswick, 3 Bailey Drive, Fredericton, New Brunswick E3B 5A3 Canada; 3 Institute for Regional Health Research, University of Southern Denmark, Odense, Denmark

**Keywords:** “Risk factors”, “Back pain”, Children, Adolescent, Young adult, Scoping review

## Abstract

**Background:**

The one-month prevalence of back pain in children and adolescents has been reported at 33, 28 and 48% at ages 9, 13 and 15 respectively. There are many suspected risk factors and triggers of back pain in young people.

**Objective:**

The purpose of this scoping review was to identify potential risk factors and potential triggers for back pain in young people. The purpose of part I was to identify potential risk factors for incident and episodic back pain in young people. Part II included all eligible studies with unclear or mixed types of back pain.

**Methods:**

Due to the vast number of studies on “risk factors” for back pain, a two-part scoping review of the literature was chosen as the best way to summarise the evidence. We adhered to the PRISMA-ScR guideline for scoping reviews. General potential risk factors and triggers for back pain in children and young adults (≤ 24 years) were included, incorporating physical, environmental, and/or physiological factors. A search was conducted using PubMed and Cochrane databases from inception to September 2018, limited to the English language. Within part I, and because of their importance, only the results of the studies that investigated risk factors of incident back pain and back pain episodes are presented.

**Results:**

The search identified 7356 articles, of which 91 articles were eligible for this scoping review. The majority of the eligible articles had an unclear definition of back pain (results presented in scoping review part II). There were 7 inception cohort studies included and 1 cohort study that met the criteria for part I. The most consistent risk factors for incident and episodic back pain are female sex and older age.

**Conclusion:**

Due to inconsistent ways of reporting on the type of back pain, no definitive risk factor for back pain has been identified. In general, females often report more symptoms, also for other diseases, and older age is not a useful risk factor as it merely indicates that the onset may not be in childhood. Clearly, the time has come to study the causes of back pain from different angles.

## Background

In children, back pain was once thought to be rare. However emerging evidence suggests that this is not the case [1]. The 1 month prevalence of back pain in children and adolescents has been reported at 33, 28 and 48% at ages 9, 13 and 15 respectively [2]. A recent systematic review found that there were three common patterns of low back pain (LBP) in children and adolescence. The majority of children (49–53%) reported no or low probability of LBP, a second group reported fluctuations of LBP (16–37%), and a minority (< 1–10%) repeatedly reported LBP [3]. The consequences of back pain included the taking of medication, missing class, and seeking care [4]. Additionally, children who report back pain have been found to have difficulty with certain activities such as standing in a queue, sports activities, and carrying a school bag [5]. There are many suspected risk factors of back pain for children and young adults.

It is important to distinguish between a risk factor for back pain and a factor associated with back pain [6]. A risk factor is defined by Porta [7] as *“a factor that is causally related to a change in the risk of a relevant health process, outcome, or condition. The causal nature of the relationship is established on the basis of scientific evidence and causal inference.”* Therefore, to identify a causal relationship rather than simply an association, the risk factor should be present, as a minimum, prior to the onset of the disease [6]. However, just because a factor precedes another does not automatically indicate causality [8]. According to the Bradford Hill criteria there are many tenets required to establish a causal link, namely: strength of association, consistency, specificity, temporality, biological gradient, plausibility, coherence, experiment, and analogy [8]. If a potential risk factor is measured concurrently with a disease, then the temporal association between the risk factor and the disease cannot be established, unless it is certain that the potential risk factor was there before the inception of the disease [6]. Therefore, generally, a prospective study design is needed to determine a risk factor [6].

If we define back pain as a ‘disease’, then the disease onset is probably the first instance of back pain [6]. An episode of back pain is an event of back pain, once this ‘disease’ has occurred, and it is a part of the relapsing and remitting nature of the ‘disease’, characterised by periods of back pain and pain-free periods. A risk factor is one that causes the ‘disease’ of back pain (marked by the first time back pain occurs) compared to a trigger, which could lead to an episode of back pain. It is possible a risk factor could also be a trigger, but not necessarily. For example, those with a genetic predisposition could be prone to develop the ‘disease’ of back pain, then a trigger for an episode could be a particular movement into an awkward position [6].

Thus to study the ‘disease’ of back pain and to identify risk factors of incident back pain, with an established temporal relationship, an inception cohort is needed [7].

Some systematic reviews have endeavoured to identify the potential risk factors of back pain in children and young adults [9–19]. Many of these located reviews focused on specific potential risk factors such as schoolbags [15], computer use [13], puberty [14], weight status [17], smoking [18], and physical activity [19]. The majority of the systematic reviews did not consider the temporal relationship between back pain and the risk factor, and combined cross-sectional studies with cohort studies and/or had unclear definitions of back pain [9, 12, 13, 15–18]. A systematic review by Ardakani et al. [6] attempted to determine if a sample of studies looking into the causes of low back pain discriminated between the back pain ‘disease’ and its episodes. They concluded that the majority of the included studies had an unclear definition of absence of low back pain at baseline and therefore cannot differentiate between back pain as the ‘disease’ and its recurring episodes [6]. Only one located systematic review by Hill and Keating [10] planned to consider the first episode of low back pain. They included only prospective studies, which they stated studied the first episode of low back pain [10]. However, half of the included articles did not actually assess the first episode of back pain and instead had unclear types of back pain, providing information on studies including first ever, episodic and ongoing back pain.

Due to the vast number of studies on risk factors for back pain, we undertook a scoping review to summarise current evidence.

The purpose of this scoping review was to identify potential risk factors and potential triggers for back pain in young people. Within this article (Part I) we included only studies that investigated risk factors (with an established temporal relationship) for incident back pain (back pain defined as the ‘disease’) and back pain defined as episodes. Part II includes all eligible studies with unclear or mixed definitions of back pain.

## Methods

We conducted a scoping review based on established guidelines [20]. A review protocol was not included in a registry and, as this was a scoping review, we did not formally rate quality including risk of bias of each article. We began with the broad question of: *what are the potential risk factors and potential triggers for back pain in childhood and young adulthood?*

### Eligibility criteria

Studies were included if they reported on any potential risk factors for pain in the thoracic and/or lumbar spine (back pain) with the majority of participants less than 25 years old at baseline. General potential risk factors and triggers for back pain in children, adolescents, and young adults up to the age of 24 years, including physical, environmental, and/or physiological factors were considered. The age classification is based on the MeSH definition of a young adult (19–24 years). Additionally, the contemporary definitions of adolescence includes young adulthood (10–24 years) [21]. We identified original peer-reviewed studies in English from any country of origin and included cohort studies, inception cohort studies and retrospective studies. Within part I, only studies that studied risk factors of incident back pain (back pain the ‘disease’) and back pain episodes were included. Therefore, for incident back pain a clear definition of the back pain that included a life-time absence of back pain at baseline was required. For episodic back pain, a clear definition of back pain with pain-free periods was required, to be able to capture recurrent back pain.

### Search strategies

**Fig. 1:**
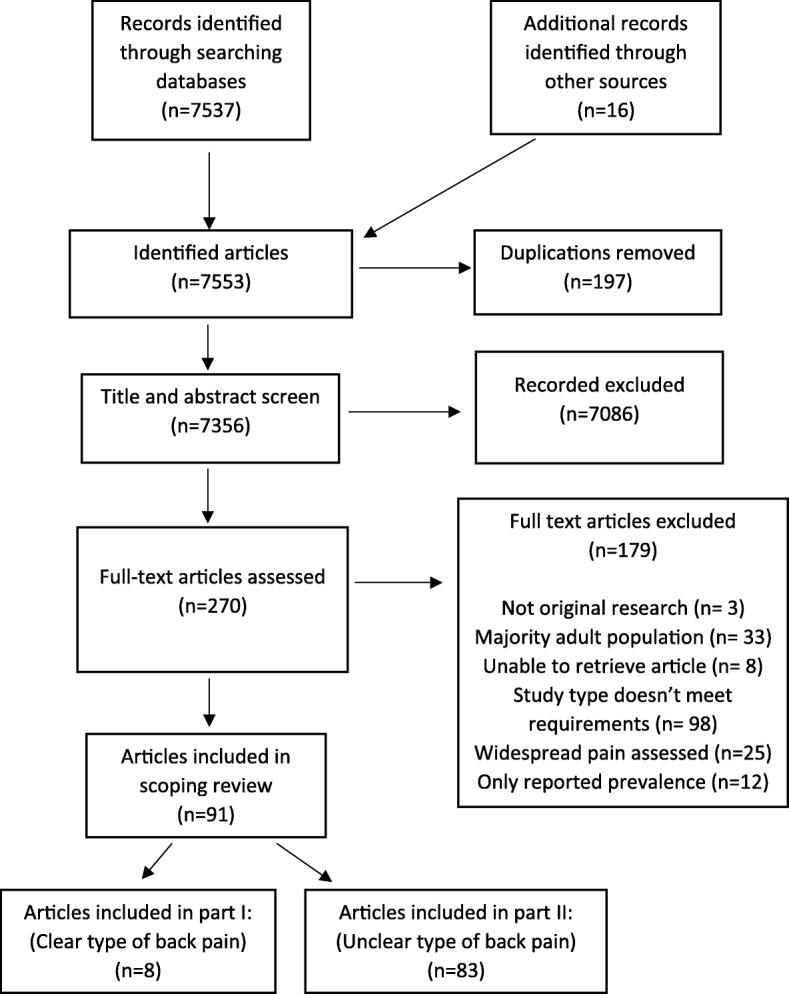
Final study selection flow diagram

A search was conducted using PubMed and Cochrane databases from inception to September 2018, limited to only English language peer-reviewed articles. In addition, reference lists of included papers and located systematic reviews were searched to identify other potentially suitable studies. There was no attempt to contact authors to identify additional sources. The full search strategy is listed in Additional file 1. Search results were imported into bibliographic management software and duplicates discarded. Results of the search were reported as per the PRISMA flow diagram (Fig. 1).

### Study selection, data charting and synthesis of results

Titles, abstracts, and full-text articles were screened by one researcher (AB) twice, once in March 2018 and then repeated in September 2018 against the inclusion criteria. The second search identified four additional articles. Another researcher (BW) verified the study selection (titles, abstracts, and full-text screen) for accuracy. One full-text article was queried, justifications provided, and full consensus was met through discussion.

Calibration of the data charting forms was conducted by two researchers (AB and CLY). One researcher (AB) piloted the form on three studies and this was verified by another researcher (CLY). This was an iterative process in which there were many changes during each round. Any disagreements were resolved by a third researcher (BW).

One researcher (AB) independently charted the data (data extraction in scoping reviews [22]) using the evidence tables. Another researcher (BW) verified the data charting for accuracy. The second reviewer had ten queries which were resolved through discussion and consensus leading to five minor changes; involvement of a third reviewer was not needed. The results were summarised reporting the number of times a risk factor was investigated, the number of times it was found to be associated with back pain, and if there was an association, the strength of this association. If a study had multiple estimates for the same risk factor, the most adjusted estimate was extracted.

Clarity of definition of back pain was assessed in each study with a summative score. Individual points were given if there was a clear description of the area of back pain, a clear reporting of the recall period, a clear definition of the type of back pain, and if there was an attempt to collect valid data (maximum four points). These scores are reported in additional files.

Data were synthesised by risk factors and further, by study design. This includes inception cohort studies reporting factors that were longitudinally associated with back pain (risk factors of back pain) and cohort studies reporting factors that were longitudinally associated with back pain episodes.

## Results

### Study selection

The database searches identified 7537 articles and 16 additional articles were identified from searching of the relevant references lists. A total of 91 articles were eligible for inclusion in this review. In all, 83 studies were excluded for part I as they did not have a clear definition of back pain or document the absence of back pain among participants at baseline. These studies were included in part II of this review. Thus, data from 8 articles were included in the current review (Fig. 1).

### Study characteristics and synthesis of results

There were 7 inception cohort studies reviewed [23–29]. These studies identified risk factors for the onset of the first episode of back pain [23–29]. Risk factors included sex, age, socioeconomic status, height, psychosocial factors, body mass index (BMI), muscle strength, physical activity, and smoking. All study populations included both males and females. The median ages of the study populations ranged from 10 to 21 years of age. Follow up periods ranged from 1 to 8 years. Charts of the summary of findings are seen in Additional file 2.

There was only one study on episodic/recurrent back pain [30]. Charts of the summary of findings are seen in Additional file 3.

### Sex

Six inception cohort studies tested sex as a potential predictor of back pain [24–29], of which two reported that females had an increased incidence of back pain [24, 29], one reported a higher incidence in males [25], and three studies found no association [26–28] (Table 1). One cohort study tested sex as a potential predictor of back pain episodes and found females had an increased prevalence of back pain [30] (Table 2).

**Table 1:** Inception cohorts: summary of risk factors for back pain the ‘disease’

Variable	Number of studies	Number of studies: increased risk	Number of studies: decreased risk	Number of studies not significant	Strength of association (95%CI)
Female sex	6	2	1	3	*Negative association:* OR 0.4 (0.3, 0.8) [25] (c) *Positive association:* OR 1.5 (1.3, 1.7) [29] (c) OR 1.8 (1.1, 3.1) [24]
Older Age	4	3	0	1	OR 2.1 (1.2, 3.7) [25] (c) OR 1.2 (1.1, 1.3) (boys) [24] OR 3.4 [27] (graph interpretation, c)
Increased physical activity	3	2	0	1	RR 1.4 (1.1, 1.9) [23] OR 2.3 [27] (graph interpretation, c)
Psychosocial	2	2	0	0	Dysfunctional coping: OR 1.4 (1.1, 2.0) (boys) [24] Anxiety sensitivity: OR: 1.5 (1.1, 2.0) (boys) [24] Somatosensory amplification: OR 1.8 (1.0,3.1) (girls) [24] Psychological distress: OR 1.9 (1.1, 3.2) [26] Emotional or behavioural disorders: OR 1.9 (1.0, 3.4) [26]
Socioeconomic	1	1	0	0	Lower parental education: OR 1.7 (1.1, 2.8) [26]
Increased growth	1	1	0	0	Increased growth spurt one SD (4.3 cm) 11–14 yr: OR 1.3 (1.1, 1.7) [28]
Muscle strength	1	1	0	0	Increased back flexor strength OR 2.8 [27] (graph interpretation, c)
Smoking	1	1	0	0	Heavy smoking: OR 1.9 (1.1, 3.1) [26]
Increased BMI	2	0	0	2	NA
Illness	1	0	0	1	NA

*OR* Odds ratio, *RR* Relative risk (c): parameter measure calculated from the provided results within study i.e. percentages converted to odds ratios, *NA* Not applicable (no significant results), *BMI* Body mass index

**Table 2:** Cohort studies: summary of risk factors for back pain episodes

Variable	Number of studies	Number of positive	Number of negative	Number not significant	Strength of association
Female sex	1	1	0	0	OR 2.1 (1.9, 2.5) [30]
Older Age	1	1	0	0	OR: (index 9 yr boy) 2.5 (1.5, 4.1) (13 yr boy), 3.2 (1.9, 5.3) (14 yr boy), 3.1 (1.8, 8.2) (15 yr boy), 3.0 (1.8, 5.2) (16 yr boy), 3.5 (1.9, 6.3) (17 yr boy), 2.4 (1.4, 4.1) (10 yr girl), 3.4 (2.1, 5.7) (11 yr girl), 4.6 (2.8, 7.5) (12 yr girl), 5.6 (3.4, 9.2) (13 yr girl), 5.4 (3.3, 8.9) (14 yr girl), 6.7 (4.1, 11.2) (15 yr girl), 6.7 (4.0, 11.3) (16 yr girl), 7.5 (4.2, 13.2) (17 yr girl) [30]

*OR* Odds ratio

### Age

Four inception cohorts tested age as a potential predictor of back pain [24–27], of which three found older age had an increased risk of back pain [24, 25, 27], and one found no association [26] (Table 1). One of these studies found age as a risk factor for back pain in males but it not in females [24], whereas another found the incidence of back pain to increase more with age in males than in females [25]. One cohort study tested age as a potential predictor of back pain episodes and found older age had an increased prevalence of back pain [30] (Table 2).

### Physical activity

Three inception cohort studies tested the relationship between physical activity and back pain [23, 26, 27]. Of these, two found that increased physical activity led to a higher incidence of back pain [23, 27], whereas one found no association [26] (Table 1). One of these studies only found this relationship with a high level of vigorous physical activity [23].

### Psychosocial factors

Two inception cohorts tested psychosocial factors as potential predictors of back pain [24, 26]. Both studies found that those with certain psychosocial factors had an increased incidence of back pain [24, 26]. Those factors included dysfunctional coping [24], anxiety sensitivity [24], somatosensory amplification [24], psychological distress [26], and emotional disorders or behavioural disorders [26] (Table 1).

### Socioeconomic status

One inception cohort tested parental education as a potential predictor of back pain and found lower parental education led to an increased incidence of back pain [26] (Table 1).

### Increased growth

One inception cohort tested increased growth as a potential predictor of back pain and found that an increased growth spurt by one standard deviation more (4.3 cm) from 11 to 14 years of age led to an increased incidence of back pain [28] (Table 1).

### Muscle strength

One inception cohort tested muscle strength as a potential predictor of back pain and found that those with an increased back flexor strength had an increased incidence of back pain. However, the study did not define what percentage of increased strength [27] (Table 1).

### Smoking

One inception cohort tested smoking status as a potential predictor of back pain and found that heavy smokers in young adulthood had an increased incidence of back pain [26] (Table 1).

### Anthropometric parameters (BMI)

Two inception cohorts tested increased BMI as a potential predictor of back pain and found no significant relationship with being in a higher BMI percentile and back pain [26, 28] (Table 1).

### Systemic/illnesses

One inception cohort tested having a chronic medical condition as a potential predictor of back pain and found no significant relationship. Chronic medical conditions were collated together and were very varied, including conditions such as: asthma, heart problems, epilepsy, cancer, diabetes, missing fingers, blindness, and “muteness” [26] (Table 1).

## Discussion

### Overall summary of risk factors or triggers for back pain

Considering the literature included in this review within part I, the factors that were found to be the most commonly investigated potential risk factors for incident back pain are female sex and older age. Based on the one study that studied episodic back pain, the potential triggers are also female sex and older age. Other factors that were identified as potential risk factors are physical activity and psychosocial factors. Consistently there was no association or a weak association noted for body mass index, height, muscle strength, smoking, and systemic/illness factors.

### Compared to previous literature

Previous systematic reviews have found similar results. Female sex [12, 16, 31] and older age [9, 12, 31] are the most frequently found risk factors for back pain during childhood and adolescence. The findings that females seem to be more at risk of back pain has been hypothesized to be due to differences in pain modulation due to oestrogen [32].

### Limitations of the current literature

The major limitations of the current literature are that the majority of studies did not adequately define back pain (incident, episodic or ongoing backpain) and the absence of back pain at baseline (Additional files 4 and 5). To identify a causal relationship, the risk factor should be present prior to the onset of the disease [6]. When studying children, there is also the question of potential memory decay, particularly when asking about the prior presence of back pain.

### Limitations of this review

This scoping review has some limitations. In accordance with PRISMA-ScR guidelines one researcher independently screened and conducted data charting, with a second researcher verifying the study selection and data charting for accuracy. However, while this method complies with the guidelines for scoping reviews it is not as rigorous as methods required for systematic review. Also, as complying with the guidelines for scoping reviews, there was no formal critical quality assessment of the included articles. Finally, only two key databases were searched, and articles were limited to the English language.

### Recommendations for future research

Future studies should follow the population from early life and capture the proposed risk factors before the onset of back pain. They should also consider the sequence of events in the causal pathway and test their hypotheses with appropriately designed longitudinal studies and appropriate analyses. They should also have a clear and consistent definition of back pain, ideally measured through a validated questionnaire. Finally, future research should concentrate on potentially modifiable risk factors.

## Conclusion

Due to inconsistent ways of reporting on the type of back pain, only a limited number of risk factors for back pain in childhood and young adulthood have been identified. Risk factors identified were predominantly biological. The most commonly investigated risk factors for back pain the ‘disease’ and back pain episodes are female sex and older age towards adolescence and young adulthood. In general, females often report more symptoms, also for other diseases, and older age is not a useful risk factor as it merely indicates that the onset may not be in childhood. Continued studies of similar approach seem not to be useful. Clearly, the time has come to study the causes of back pain from different angles.

## Supplementary information

**Additional file 1.** Search strategies used for the literature search. The full search strategy for PubMed and Cochrane databases.

**Additional file 2.** INCEPTION COHORT STUDIES reporting factors that are longitudinally associated with back pain. Table summarising each included inception cohort study.

**Additional file 3.** COHORT STUDIES reporting factors that are longitudinally associated with back pain episodes. Table summarising included cohort study.

**Additional file 4.** Clarity of definitions of Back pain: Inception Cohort studies. Table summarising the clarity of the definitions of back pain in included inception cohort studies.

**Additional file 5.** Clarity of definitions of Back pain: Cohort studies. Table summarising the clarity of the definitions of back pain in included cohort study.

## Data Availability

Not applicable.

## Abbreviations

BMI: Body mass index
CI: Confidence intervals
LBP: Low back pain
N: Number of participants
NA: Not applicable
OR: Odds ratio
RR: Relative risk
