# Supplementary material for: Potential risk factors and triggers for back pain in children and young adults. A scoping review, part I: incident and episodic back pain
Source: Chiropr Man Therap. 2019 Nov 19;27:58. doi: 10.1186/s12998-019-0280-9 (PMC6862727; doi:10.1186/s12998-019-0280-9)
Supplement: Supplementary file 1 — Additional file 1. Search strategies used for the literature search. The full search strategy for PubMed and Cochrane databases. [file 12998_2019_280_MOESM1_ESM.pdf]

Additional file 1: Search strategy used for the literature search

Databases: PubMed, Cochrane Database. Database search from inception to September 2018, limited to English articles.

PubMed Search:

1. “young adult”
2. “adolescen*”
3. “teenager”
4. “juvenile”
5. “child*”
6. 1 OR 2 OR 3 OR 4 OR 5
7. “low back pain”
8. “back pain”
9. “mid back pain”
10. 7 OR 8 OR 9
11. “risk”
12. “risk factor”
13. “causality”
14. “epidemiological factor”
15. “indicators”
16. “prognostic”
17. “cause”
18. “comorbidities”,
19. “prevalence”
20. “incidence”.
21. 11 OR 12 OR 13 OR 14 OR 15 OR 16 OR 17 OR 18 OR 19 OR 20
22. 6 AND 10 AND 21

Cochrane Search:

1. young adult OR adolescen* OR teenager OR juvenile OR child*
2. low back pain OR back pain OR mid back pain
3. risk OR risk factor OR causality OR epidemiological factor OR indicators OR prognostic OR cause OR comorbidities OR prevalence OR incidence
4. S1 AND S2 AND S3
