# Supplementary material for: Potential risk factors and triggers for back pain in children and young adults. A scoping review, part I: incident and episodic back pain
Source: Chiropr Man Therap. 2019 Nov 19;27:58. doi: 10.1186/s12998-019-0280-9 (PMC6862727; doi:10.1186/s12998-019-0280-9)
Supplement: Supplementary file 2 — Additional file 2. INCEPTION COHORT STUDIES reporting factors that are longitudinally associated with back pain. Table summarising each included inception cohort study. [file 12998_2019_280_MOESM2_ESM.pdf]

Additional file 2: INCEPTION COHORT STUDIES reporting factors that are longitudinally associated with back pain.

Back pain, sample characteristics and associations between back pain and risk factors of back pain. Inception Cohorts

Reference	Back Pain					Characteristics of study sample			Significant positive (+) or negative (-) associations with back pain					
**Reference (year of pub), country, pop size**	**MBP**	**LBP**	**Mix**	**?**	**Clear definition of BP (x/4) (Additional file 4)**	**Age range at baseline**	**Sex**	**No. of follow ups Follow-up period**	**Female**	**Male**	**Age**	**Height**	**Socioeconomic factors**	**Significant estimates (95% CI)**
[23] Aartun, (2016), Denmark, 144	X	X	X		**4/4**	11-13	Both	1<br>2 years	(NT)	(NT)	(NT)	(NT)	(NT)	
[24] Barke, (2014), Germany, 2040				X	2/4	9-14	Both	1<br>1 year	+	(NT)	+	(NT)	(NT)	Female: OR 1.8 (1.1-3.1)<br>Age: OR 1.2 (1.1-1.3)
[25] Burton, (1996), England, 216		X			3/4	11	Both	4<br>1 year		+	+	(NT)	(NT)	Males: prevalence percentage boys 60%, girls 40%<br>(Female: OR 0.4 (0.3-0.8) (calculated))<br>Older age 11 yrs: 12% 15 yrs: 22%<br>(Older age: OR 2.1 (1.2-3.7) (calculated))
[26] Mustard, (2005), Canada, 1043				X	3/4	4-16	Both	2<br>4 years, 14 years	0	0	0	(NT)	+	Lower parental education: OR 1.7 (1.1-2.8)
[27] Newcomer, (1996), USA 96		X			**4/4**	10-19	Both	1<br>4 years	0	0	+	(NT)	(NT)	Older age OR 3.4 (graph interpretation)
[28] Poussa, (2005), Finland, 430		X			3/4	10-11	Both	5<br>1 year (4x), 8 years (1)	0	0	(NT)	+	(NT)	Increased growth spurt of one SD (4.3cm) from 11-14 years: OR 1.3 (1.1-1.7)
[29] Triki, (2015), Tunisia, 5958		X			3/4	18-24	Both	7<br>1 year	+		(NT)	(NT)	(NT)	Female: Female 17%, male 13%<br>(Female: OR 1.5 (1.3-1.7) (posthoc))<br>(the sex differences disappeared when looking at individual sports)
Pub: Publication, MBP: Mid-back pain, LBP: low back pain: BP: back pain, (NT): Not tested, + significant positive association, - significant negative association, 0: tested but non-significant estimate, CI: confidence interval, OR: odds ratio, SD: standard deviation, RR: relative risk, No.: number														

Back pain, sample characteristics and associations between back pain and risk factors of back pain continued. Inception cohorts

Reference	Back Pain					Characteristics of study sample			Significant positive (+) or negative (-) associations with back pain						
**Reference (year of pub), country, pop size**	**MBP**	**LBP**	**Mix**	**?**	**Clear definition of BP (x/4) (Additional file 4)**	**Age range at baseline**	**Sex**	**Follow-up period**	**BMI**	**Muscle strength**	**Psychosocial**	**Physical activity/ work**	**Smoking**	**Illness**	**Significant estimates (95% CI)**
[23] Aartun, (2016), Denmark, 144	X	X	X		**4/4**	11-13	Both	1<br>2 years	(NT)	(NT)	(NT)	+	(NT)	(NT)	High level physical activity: RR 1.4 (1.1-1.9)
[24] Barke, (2014), Germany, 2040				X	2/4	9-14	Both	1<br>1 year	(NT)	(NT)	+	(NT)	(NT)	(NT)	Dysfunctional coping: OR 1.4 (1.1-2.0) (boys)<br>Anxiety sensitivity:<br>OR 1.5 (1.1-2.0) (boys)<br>Somatosensory amplification:<br>OR 1.8 (1.0-3.1) (girls)<br>Pain catastrophizing: OR 0.5 (0.3-0.9) (girls)
[25] Burton, (1996), England, 216		X			3/4	11	Both	4<br>1 year	(NT)	(NT)	(NT)	(NT)	(NT)	(NT)	
[26] Mustard, (2005), Canada, 1928				X	3/4	4-16	Both	2<br>4 years,<br>14 years	0	(NT)	+	0	+	0	Heavy smoking: OR 1.9 (1.1-3.1)<br>Psychological distress:<br>OR 1.9 (1.1-3.0) (low), OR 1.9 (1.1-3.2) (mod/high)<br>Emotional or behavioural disorders:<br>OR 1.9 (1.0-3.4)
[27] Newcomer, (1996), USA 96		X			**4/4**	10-19	Both	1<br>4 years	(NT)	+	(NT)	+	(NT)	(NT)	Increased level of physical activity: OR 2.3 (graph interpretation)<br>Stronger back flexors: OR 2.8 (graph interpretation)
[28] Poussa, (2005), Finland, 430		X			3/4	10-11	Both	5<br>1 year (4x), 8 years (1)	0	(NT)	(NT)	(NT)	(NT)	(NT)	
[29] Triki, (2015), Tunisia, 5958		X			3/4	18-24	Both	7<br>1 year	(NT)	(NT)	(NT)	(NT)	(NT)	(NT)	

Pub: Publication, MBP: Mid-back pain, LBP: low back pain: BP: back pain, (NT): Not tested, + significant positive association, - significant negative association, 0: tested but non-significant estimate, CI: confidence interval, OR: odds ratio, SD: standard deviation, RR: relative risk, No.: number
