# Supplementary material for: Potential risk factors and triggers for back pain in children and young adults. A scoping review, part I: incident and episodic back pain
Source: Chiropr Man Therap. 2019 Nov 19;27:58. doi: 10.1186/s12998-019-0280-9 (PMC6862727; doi:10.1186/s12998-019-0280-9)
Supplement: Supplementary file 3 — Additional file 3. COHORT STUDIES reporting factors that are longitudinally associated with back pain episodes. Table summarising included cohort study. [file 12998_2019_280_MOESM3_ESM.pdf]

Additional file 3: COHORT STUDIES reporting factors that are longitudinally associated with back pain episodes.

Back pain, sample characteristics and associations between back pain and temporal precursor variables. Cohort Studies

**Reference**	**Back pain**					**Characteristics of study sample**			**Significant positive (+) or negative (-) associations with back pain**			
**Ref, (year of pub), country, pop size**	**MBP**	**LBP**	**Mix**	**?**	**Clear definition of BP (x/4) (Additional file 5)**	**Age range at baseline**	**Sex**	**No. of follow ups Follow-up period**	**Female**	**Male**	**Age**	**Significant estimates (95% CI)**
[30] van Gessel, (2011), Germany, 2025				X	2/4	9-14	Both	3<br>1 year	+		+	Females: OR 2.1 (1.9-2.5)<br>Age: 9yr boy: OR 1 (index)<br>2.5 (1.5, 4.1) (13, boy), 3.2 (1.9, 5.3) (14, boy), 3.1 (1.8, 8.2) (15, boy), 3.0 (1.8, 5.2) (16, boy), 3.5 (1.9, 6.3) (17 boy), 2.4 (1.4, 4.1) (10 girl), 3.4 (2.1, 5.7) (11, girl), 4.6 (2.8, 7.5) (12, girl), 5.6 (3.4, 9.2) (13, girl), 5.4 (3.3, 8.9) (14, girl), 6.7 (4.1, 11.2) (15, girl), 6.7 (4.0, 11.3) (16, girl), 7.5 (4.2, 13.2) (17, girl).

Pub: Publication, MBP: Mid-back pain, LBP: low back pain: BP: back pain, + significant positive association, OR: odds ratio, No.: number
