# Supplementary material for: Potential risk factors and triggers for back pain in children and young adults. A scoping review, part I: incident and episodic back pain
Source: Chiropr Man Therap. 2019 Nov 19;27:58. doi: 10.1186/s12998-019-0280-9 (PMC6862727; doi:10.1186/s12998-019-0280-9)
Supplement: Supplementary file 4 — Additional file 4. Clarity of definitions of Back pain: Inception Cohort studies. Table summarising the clarity of the definitions of back pain in included inception cohort studies. [file 12998_2019_280_MOESM4_ESM.pdf]

Additional file 4: Clarity of definitions of Back pain: Inception Cohort studies

	**Area of BP (1 point)**	**Recall period (1 point)**						**Type (1 point)**	**Severity described**	**Consequences reported**	**Attempted to collect valid data (1 point)**	**Conclusion**
**Ref (year of pub)**	**Location**	**Now**	**Past week**	**Past month**	**Past year**	**> 1 year**	**pain ever**	**-1st ever<br>-Episodic<br>-Ongoing<br>-?**		**-Seek care<br>-Downtime<br>-Disability**		**Clear definition of BP (x/4)**
[23] Aartun, (2016)	MB/LB*						X	-1st ever<br>-<br>-<br>-	No	-<br>-<br>-	Diagram used, pilot study of the questionnaire	**4/4**
[24] Barke, (2014)	?			X (6 mth)	X		X	-1st ever<br>-<br>-<br>-	No	-<br>-<br>-	NR	2/4
[25] Burton, (1996)	LB	X			X		X	-1st ever<br>-<br>-<br>-	No	-Seek care<br>-<br>-Disability	NR	3/4
[26] Mustard, (2005)	?				X		X	-1st ever<br>-<br>-<br>-	No	-<br>-<br>-Disability	Used a pre-validated questionnaire	3/4
[27] Newcomer, (1996)	LB				X		X	-1st ever<br>-<br>-<br>-	No	-Seek care<br>-Downtime<br>-	Used a pre-validated questionnaire	**4/4**
[28] Poussa, (2005)	LB				X		X	-1st ever<br>-<br>-<br>-	No	-<br>-<br>-	Diagram used	3/4
[29] Triki, (2015)	LB					X		-1st ever<br>-<br>-<br>-	No	-Seek care<br>-<br>-	NR	3/4

BP: back pain, LB: low back, MB: mid back, NR: not reported, mth: months, MB/LB*: collected data from regions separately, however reported together as spinal pain
