# Supplementary material for: Potential risk factors and triggers for back pain in children and young adults. A scoping review, part I: incident and episodic back pain
Source: Chiropr Man Therap. 2019 Nov 19;27:58. doi: 10.1186/s12998-019-0280-9 (PMC6862727; doi:10.1186/s12998-019-0280-9)
Supplement: Supplementary file 5 — Additional file 5. Clarity of definitions of Back pain: Cohort studies. Table summarising the clarity of the definitions of back pain in included cohort study. [file 12998_2019_280_MOESM5_ESM.pdf]

Additional file 5: Clarity of definitions of Back pain: Cohort studies

	Area of BP (1 point)	Recall period (1 point)						Type (1 point)	Severity described	Consequences reported	Attempted to collect valid data (1 point)	Conclusion
**Ref (year of pub)**	**Location**	**Now**	**Past week**	**Past month**	**Past year**	**> 1 year**	**pain ever**	**-1st ever<br>-Episodic<br>-Ongoing<br>-?**		**-Seek care<br>-Downtime<br>-Disability**		**Clear definition of BP (x/4)**
[30] van Gessel, (2011)	?			X (6 mth)				-<br>-Episodic<br>-<br>-	Yes	-<br>-<br>-	NR	2/4

BP: back pain, LB: low back, MB: mid back, NR: not reported, mth: months
